# Glargine versus regular insulin protocol in feline diabetic ketoacidosis

**DOI:** 10.1111/vec.13062

**Published:** 2021-05-04

**Authors:** Florian K. Zeugswetter, Nicole Luckschander‐Zeller, Sonja Karlovits, Jaquie S. Rand

**Affiliations:** ^1^ University Hospital for Small Animals of the University of Veterinary Medicine Vienna Austria; ^2^ School of Veterinary Science The University of Queensland Australia

**Keywords:** diabetes mellitus, intramuscular, potassium, saline, β‐hydroxybutyrate

## Abstract

**Objectives:**

To determine whether basal‐bolus administration of glargine insulin is a safe and effective alternative treatment compared to the standard continuous rate infusion (CRI) protocol.

**Design:**

Prospective randomized clinical trial.

**Setting:**

University teaching hospital.

**Animals:**

Twenty cats diagnosed with diabetic ketoacidosis (DKA).

**Interventions:**

The cats were block‐randomized to either a CRI protocol using regular insulin (CRI‐group; n = 10) or a basal‐bolus SC and IM glargine protocol (glargine‐group, n = 10). Baseline blood gases, electrolytes, glucose, and β‐hydroxybutyrate (β‐OHB) concentrations were measured at the time of admission and later at predefined intervals until reaching the primary endpoint of the study, defined as a β‐hydroxybutyrate concentration < 2.55 mmol/L.

**Measurements and main results:**

The main outcome measure was time (h) to resolution of ketonemia. Secondary outcome measures were time until first improvement of hyperglycemia and ketonemia, decrease of glucose to ≤13.9 mmol/L (250 mg/dL), resolution of acidosis, consumption of first meal, and discharge from hospital. Additionally, occurrence of treatment‐associated adverse events and death were compared. Seventeen cats (85%) survived to discharge, with no difference in survival between groups (*P* = 1.0). Median times to β‐OHB < 2.55 mmol/L were 42 (CRI‐group) and 30 (glargine‐group) hours, respectively (*P* = 0.114). Median times to first improvement of hyperglycemia (glargine‐group: 2 h; CRI‐group: 6 h; *P* = 0.018) and until discharge from hospital (glargine‐group: 140 h; CRI‐group: 174 h; *P* = 0.033) were significantly shorter in the glargine‐group. No significant differences were observed in any other parameter under investigation (*P* > 0.05).

**Conclusions:**

Basal‐bolus administration of glargine insulin appears to be an effective and safe alternative to the current standard CRI‐protocol for the management of DKA in cats. The positive outcomes and simplicity make it a viable option for the treatment of feline DKA.

**Abbreviations**
CRI: constant rate infusion
DKA: diabetic ketoacidosis
DM: diabetes mellitus

## 1 INTRODUCTION

Diabetic ketoacidosis (DKA) is an acute, life‐threatening metabolic complication of feline diabetes mellitus (DM), and mortality rates range from 17 to 50%.^1^, ^2^, ^3^, ^4^, ^5^, ^6^ Despite this, studies are scarce, and current treatment recommendations are mostly extrapolated from experiences in people and dogs. The 3 cornerstones of therapy are rehydration, correction of electrolyte imbalances, and insulin administration to reduce ketone and glucose concentrations. Published insulin treatment regimens include intermittent IM administration of regular insulin^2^, ^7^ or glargine,^8^ continuous rate infusion of regular insulin^1^, ^3^, ^5^, ^9^ or lispro,^1^, ^6^ the combinations of SC glargine with intermittent IM regular insulin^5^ or the combination of SC and IM glargine.^8^ The relatively small number of patients included in the studies and limited comparisons between different protocols precludes evidence‐based recommendations regarding which protocol is superior, and additional research is required. The use of the insulin analog glargine is of special interest as it is considered an insulin of first choice in diabetic cats,^10^, ^11^ has a long half‐life when injected subcutaneously,^12^ and elicits comparable effects to regular insulin when given intravenously^13^, ^14^ or intramuscularly^15^ in people. In a retrospective study of 15 cats with DKA, SC glargine administration was started immediately after the diagnosis of DKA and combined with IM glargine injections as required.^8^ As the survival rate was 100% and hospitalization time was short, this basal‐bolus treatment was considered a feasible alternative to traditional treatment regimens. Several studies in ketoacidotic diabetic human patients have shown that IM^17^ or even SC^18^, ^19^ bolus treatment protocols are safe and an effective treatment option. The objective of this prospective block‐randomized study was to compare the basal‐bolus glargine regimen with a regular IV insulin protocol and to demonstrate its utility in the treatment of feline DKA.

## 2 MATERIALS AND METHODS

### 2.1 Case selection

Client‐owned cats with naturally occurring DKA admitted to the University of Veterinary Medicine Vienna between January 2014 and October 2017 were considered for inclusion. Only cats with a patient history consistent with diabetes mellitus (polyuria and polydipsia with or without polyphagia or weight loss), at least 2 clinical signs consistent with DKA (mentation score ≥1,^20^ vomiting or anorexia), blood glucose concentration above the renal threshold (15 mmol/L [270 mg/dL]),^21^ blood β‐hydroxybutyrate (β‐OHB) concentration > 2.55 mmol/L,^22^ and a metabolic high anion gap (AG) acidosis (pH < 7.27 and an AG > 20.6 mmol/L), ^23^ were included. Criteria for exclusion included International Renal Interest Society (IRIS) kidney failure greater than stage 3 (creatinine > 431 μmol/L [> 4.9 mg/dL]),^24^ congestive heart failure, a mentation score 4, and if the treating clinicians did not adhere to the prescribed protocol. Mentation was judged according to the mentation score described by Hayes et al^20^ (0 = normal; 1 = able to stand unassisted, responsive but dull; 2 = can stand only when assisted, responsive but dull; 3 = unable to stand, responsive; 4 = unable to stand, unresponsive).

### 2.2 Therapy and monitoring

At or near the time of presentation, a complete medical history and physical examination was performed, and blood was collected from the cephalic or jugular vein. Abdominal ultrasound was obligatory, whereas other diagnostic tests such as thoracic radiographs were performed according to the clinician's discretion. Whenever possible, urine was collected by cystocentesis. Glucose (capillary blood) and β‐OHB (venous blood) were measured every 2 and 6 hours, respectively. A human glucometer^a^ and a ketometer,^b^ validated for the use in cats,^22^, ^25^ were used from 2014 to 2015 and thereafter replaced by a validated gluco‐/ketometer^c^ ^,^ ^d^ marketed for cats. Venous blood gas analysis^e^ (temperature corrected ph‐stat) and phosphorus concentrations^f^ were evaluated every 6 and 24 hours, respectively. Magnesium was measured in case of refractory hypokalemia and supplemented with 0.75 mmol/kg/24 h if indicated. All cats received standard care for DKA,^16^, ^26^ including IV 0.9% NaCl (fluid deficits: % dehydration × bodyweight × 1000 = mL/24 h; maintenance: 2 mL/kg/h, ongoing losses: estimated), potassium (added to 250 mL fluid bag at various amounts dependent on potassium concentration; Table 1), and phosphorus (25% of potassium as potassium‐phosphate; not in cats with phosphate > 1.6 mmol/L [5 mg/dL]). If indicated after cardiovascular system evaluation on admission, intermittent boluses (10‐15 mL/kg) of 0.9% NaCl without potassium supplementation were administered over 15 to 20 minutes. Antiemetics^g^ and gastric protectants^h^ were also administered and, where indicated, antimicrobials (amoxicillin as first choice) and pain management (opioids). Additional phosphorus was added to the infusions in cats with more severe hypophosphatemia (≤0.49 mmol/L [1.5 mg/dL]).^27^ Bicarbonate administration was at the discretion of the veterinarian at a pH < 7 in combination with a bicarbonate concentration < 11 mmol/L and mandatory at a pH < 6.9.^28^ If administered, it was required to be discontinued at pCO_2_ (venous) concentrations > 0.51 kPa (38 mm Hg) to avoid paradoxical cerebral acidosis. For insulin treatment, the cats were randomly assigned (block randomization with 4 cats in each block) to 1 of 2 treatment groups: CRI‐group (constant rate regular insulin infusion group) or glargine‐group (intermittent SC/IM glargine).

**TABLE 1:** Potassium supplementation protocol for constant rate infusion (not intended for bolus treatment)

Blood potassium concentration (mmol/L)	Potassium concentration (mmol/L) required in IV fluids*
>5.0	0
4.1‐5.0	20
3.6‐4	30
3.1‐3.5	40
2.6‐3	50
2.1‐2.5	60
≤2	80

* Recommended infusion rate < 0.5 mmol/kg/h.

In the CRI‐group, 1.1 units per kg body weight of regular insulin^i^ was added to a 250 mL bag of 0.9% saline, and the first 50 mL was drained out to allow for insulin absorption by the plastic tubing. This fixed‐weight basal insulin infusion was initiated 2 hours after starting rehydration with a constant rate of 10 mL/h (∼0.05 units/kg/h) and adjusted every 2 hours as required to achieve a decrease in glucose concentration of 2 to 3 mmol/L/h (36‐54 mg/dL). In the glargine‐group, all cats received a bolus of 2 SC units of insulin glargine^j^ irrespective of body weight concurrently with starting rehydration and 1 IM unit/cat 2 hours thereafter. IM injections (1 unit/cat) were subsequently repeated every 4 hours if glucose was > 13.9 mmol/L (250 mg/dL), and SC glargine was continued every 12 hours with 0.25 units/kg (based on estimated ideal body weight)^29^ rounded to the next half or whole unit. The primary aim in both protocols was to achieve a blood glucose concentration < 13.9 mmol/L (250 mg/dL). At that point, intensive insulin therapy was continued at 10 mL/h, but IV fluids were changed to a 2.5 to 5% glucose‐containing solution and adjusted to keep blood glucose concentration between 10 and 13.9 mmol/L (180 and 250 mg/dL). Based on recommendations in people,^19^ insulin administration was only decreased (to 5 mL/h) or stopped (at the discretion of the clinician in charge) in cats with glucose < 4.44 mmol/L (80 mg/dL), despite 5% glucose infusions at the calculated infusion rate (see above). No glucose was added to the insulin infusion. In cats with inappetence for more than 3 days, a nasoesophageal feeding tube was placed, and the cats were fed 4 times/d with a liquid diet^k^ using standard estimation of resting energy requirements (70 x [body weight in kg]^0.75^). When the cats were appropriately hydrated, started to eat spontaneously, and β‐OHB was < 2.55 mmol/L, the cats in both groups were changed to an exclusively SC insulin regimen (glargine q 12 h with 0.25‐0.5 units/kg [based on estimated ideal body weight and blood glucose concentration]).^27^

The primary aim of the study was to compare time to plasma β‐OHB concentration < 2.55 mmol/L between the insulin groups. Time 0 was defined as the time an IV catheter was placed and infusion therapy was started. Secondary outcome measures were time (h) to improvement of hyperglycemia and ketonemia, glucose ≤13.9 mmol/L [250 mg/dL]), resolution of acidosis (pH < 7.27), consumption of first meal, and discharge from hospital. Improvement of hyperglycemia and ketonemia was defined as a reduction of glucose and β‐OHB concentrations greater than the mean coefficients of variation of these solutes at high concentrations (CV% glucose 1.6%, CV% β‐OHB 6.4%)^l^ measured with the gluco‐/ketometer.^c^ Additionally, the occurrence of associated adverse events such hypoglycemia (glucose < 3.7 mmol/L [67 mg/dL]; reference interval, 3.7‐10.5 mmol/L [67‐189 mg/dL]),^30^ severe hypophosphatemia (phosphate < 0.49 mmol/L [1.5 mg/dL]; reference interval, 0.8‐1.6 mmol/L[2.5‐5 mg/dL]),^27^ severe hypokalemia (potassium < 3 mmol/L [< 3 mEq/L]; reference interval, 3.5‐5 mmol/L [3.5‐5 mEq/L]),^31^corrected hyperchloremia (chloride > 123 mmol/L [> 123 mEq/L]; reference interval, 117–123 mmol/L [117‐123 mEq/L]),^32^ hypernatremia (> 164 mmol/L [> 164 mEq/L]; reference interval, 142–164 mmol/L [142‐164 mEq/L]), severe or moderate hypothermia (< 34°C or 34–36.5°C),^33^ and bradycardia (< 120/min) were compared, and cause of death was determined where possible. Cats were discharged from the hospital when there was no vomiting, the cat was eating, β‐OHB was < 2.55 mmol/L, and the cat's condition was assessed by the clinician as suitable for discharge to the owners. Presence of acidosis did not preclude the cat being discharged if it was caused by concurrent disease (eg, renal failure).

The study protocol was approved by the Austrian Ethical and Animal Welfare Committee (BMWF‐68.205/0004‐II/3b/2014).

### 2.3 Statistical methods

All analyses were performed using statistical software .^m^ Due to the small number of cats in both groups, and because many data were not normally distributed (visual evaluation and Kolmogorov–Smirnov test), all variables are presented as median (range), and data were compared between the groups using the non‐parametric Mann–Whitney *U*‐test. Categorial variables were compared using Fisher's exact test. Kaplan–Meier plots were generated and evaluated with the log‐rank test. Cats that did not achieve a specific outcome (eg, discharge from hospital, consumption of first meal, or glucose < 13.9 mmol/L [< 250 mg/dL]) were censored for the purpose of Kaplan–Meier analysis. Associations between pair‐wise variables were examined using Spearman's rank‐order correlation (*r*_SP_) and were classified as strong (*r*_SP_ ≥0.5‐1), moderate (*r*_SP_ = 0.3‐0.5), or weak (*r*_SP_ < 0.3), respectively. Based on a priori knowledge of the expected direction of change in outcome measures, correction for multiple analyses was not applied, and significance was set at a *P*‐value of < .05.

## 3 RESULTS

In the study period, 37 cats with diabetes mellitus and clinical suspicion for ketoacidosis were evaluated. Seventeen cats were excluded because of a pH > 7.27 (n = 9), congestive heart failure (n = 4), and stage 4 kidney failure (n = 2). Post hoc exclusions occurred because of initiation of the wrong protocol (n = 1) and variation of the protocol (n = 1). The remaining cats (n = 20) that met all criteria for inclusion were block‐randomized to 1 of the 2 groups (10 cats per group). Except for 1 Manx (CRI‐group) and 1 Russian Blue cat (glargine‐group), all cats were domestic shorthair cats. Demographic characteristics including age, sex, weight, body condition score, and baseline variables were not significantly different between the groups (Table 2). There were 6 and 3 cats with newly diagnosed and untreated DM in the CRI and glargine‐group, respectively (*P* = 0.37). One cat in each group had been pretreated with prednisolone, 2 cats in the CRI‐group and 4 cats in the glargine–group were deemed fractious (*P* = 0.629). All but 1 cat (score 2) in the CRI‐group and 3 cats (all score 2) in the glargine‐group had a mentation score of 1 at presentation (*P* = 0.582).

**TABLE 2:** Signalment and baseline data of 20 cats diagnosed with diabetic ketoacidosis and block‐randomized to 1 of 2 treatment protocols

	CRI‐group (n = 10)	Glargine‐group (n = 10)	*P*‐value
Age (y)	9.5 (5‐15)	10.5 (4.5‐15)	0.796
Gender			
Male	4 (all neutered)	6 (all neutered)	
Female	6 (1 intact)	4 (all neutered)	0.656
Breed			
Domestic shorthair	9	9	
Manx	1	0	
Russian Blue	0	1	
Body weight (kg)	3.4 (2.8‐6.5)	4.2 (2.9‐5.4)	0.529
Body conditioning score (1‐5)	2 (1‐5)	3 (1‐4)	0.126
Known diabetes mellitus	4	7	0.37
Mentation score (0‐4)*	1 (1‐2)	1 (1‐2)	0.582
Heart rate (/min)	154 (130‐186)	164 (144‐200)	0.089
Body temperature (°C)	37.6 (34.9‐39.8)	38.3 (37‐40.5)	0.393
Glucosuria (dipstick)	8 of 8	9 of 9	1.000
Ketonuria (dipstick)	7 of 8	7 of 9	1.000
HCT (L/L)	0.31 (0.24‐0.49)	0.385 (0.29‐0.48)	0.123
Total plasma protein (g/L)	78 (62‐100)	81 (71‐118)	0.315
Glucose (mg/dL)	421 (277‐619)	421 (276‐589)	1.000
Glucose (mmol/L)	23.4 (15.4‐34.4)	23.4 (15.3‐32.7)	1.000
β‐Hydroxybutyrate (mmol/L)	7.6 (4.4‐8)	6.3 (4‐8)	0.436
Creatinine (mg/dL)	1.4 (0.6‐1.98)	1.1 (0.6‐1.6)	0.436
Creatinine (μmol/L)	123.8 (53‐175.1)	97.3 (53‐141.5)	0.436
Alanine aminotransferase (U/L)	260 (73‐1,036)	214 (102‐427)	0.481
Phosphorus (mmol/L)	1.15 (0.52‐2.96)	1.1 (0.52‐1.9)	0.549
Specific gravity urine	1.023 (1.007‐1.045)	1.019 (1.007‐1.050)	0.918
pH	7.19 (7.06‐7.27)	7.2 (6.94‐7.27)	1.000
Bicarbonate (mmol/L)	10.4 (6.9‐13.8)	10.8 (5‐17.8)	0.853
Anion gap (mmol/L)	31 (22‐40)	34 (22‐37)	0.912
Potassium (mmol/L)	2.5 (1.8‐4.6)	3.15 (1.5‐4.1)	0.853
Sodium (mmol/L)	149 (138‐169)	152 (123‐172)	1.000
Chloride (mmol/L)	109 (101‐122)	111 (94‐121)	0.912
Chloride corrected (mmol/L)	113 (110‐118)	113 (107‐118)	0.912

CRI, continuous rate infusion.

Data are presented as median (range). *P* < 0.05 is considered significant.

* According to Hayes et al.^20^

One cat in the glargine‐group with hepatic failure and bacterial cystitis was euthanized due to financial constraints on day 2 before reaching the primary endpoint of the study. Histopathology was not performed.

In the CRI‐group, 1 cat died on day 7, likely as a consequence of liver failure (icterus, alanine aminotransferase [ALT], 1000 U/L), and the owners elected euthanasia on day 5 for another cat with worsening mentation (mentation score 3). Postmortem examination was not performed in the first cat, and histopathology in the second cat revealed severe multifocal purulent myocarditis, pancreatitis, insulitis, focal hepatic necrosis, and a thyroid adenoma.

Seventeen cats survived to discharge (85%, CRI‐group, n = 8/10; 90%, glargine‐group, n = 9/10). One of these cats (CRI‐group) was euthanized within 1 week after discharge from hospital because of lethargy and reluctance to eat. Histopathology revealed severe hepatic lipidosis, severe interstitial nephritis, moderate purulent pancreatitis, 2 small duodenal ulcerations, and 2 deep tracheal ulcerations associated with fungal infestation.

One cat in the CRI‐group received 1 bolus, and 1 cat in the glargine‐group received 2 boluses of isotonic crystalloids before initiating CRI. The cat in the CRI‐group additionally received hydroxyethyl starch (2 mL/kg over 5 minutes) for volume resuscitation. Although 2 cats in the glargine‐group had a pH < 7.00 (pH = 6.94 and 6.99) at presentation, no cat had a pH < 6.9, which was the criteria for mandatory bicarbonate therapy. Both cats survived to discharge without bicarbonate administration. One cat in the glargine‐group received magnesium sulfate. A nasoesophageal feeding tube was placed in 8 cats (CRI‐group, n = 5; glargine group, n = 3; *P* = 0.65). Two cats in the CRI‐group developed furosemide‐responsive respiratory distress and hydrothorax, likely caused by overhydration. Eight (CRI‐group) and 6 (glargine‐group) cats were treated with antibiotics, respectively (*P* = 0.629).

**FIGURE 1:**
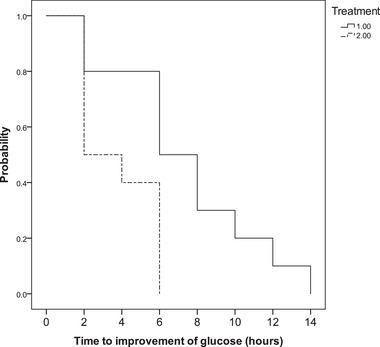
Kaplan–Meier curves of cats treated with the CRI‐ (treatment 1; n = 10) or the glargine‐protocol (treatment 2; n = 10) showing the time (h) from the start of treatment until an improvement of hyperglycemia (decrease in glucose concentration of greater than 1.6% of the basal concentration) was first documented. The curves of the 2 groups were compared using the log‐rank (Mantel–Cox) test (*P *= 0.018)

**FIGURE 2:**
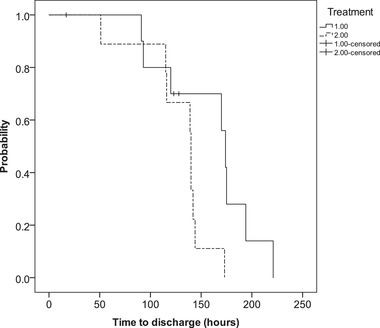
Kaplan–Meier curves of cats treated with the CRI‐ (treatment 1; n = 10) or the glargine‐protocol (treatment 2; n = 10) showing the time (h) from the start of treatment until discharge from the hospital. The curves of the 2 groups were compared using the log‐rank (Mantel–Cox) test (*P* = 0.033)

The time until β‐OHB decreased to < 2.55 mmol/L was comparable between the groups (30 [12‐42] vs 42 [18‐102] h; *P* = 0.092). The glargine‐group had significantly shorter median times until first improvement of hyperglycemia, defined as > 1.6% decrease from baseline (2 [2‐6] h vs 6 [2‐14] h; *P* = 0.018, Figure 1) and until discharge from hospital (140 [51‐173] h vs 174 [91‐221] h; *P* = 0.033, Figure 2, Table 3). No differences were observed in any other parameter under observation (Table 2). None of the cats exhibited clinical signs suggestive of hypoglycemia. The change of HCT from baseline to discharge was moderately correlated (*r*_SP _= 0.5, *P* = 0.021) with the lowest phosphorus concentration determined during treatment. The choice of insulin protocol had no influence on changes of HCT, total protein, ALT, creatinine, or weight (Table 3). In addition to the first IM insulin injection in the glargine‐group, a median (range) of 3 (0‐11) additional IM glargine injections were administered.

**TABLE 3:** Outcome for 20 cats diagnosed with diabetic ketoacidosis and treated with the CRI‐ or the glargine‐protocol

	CRI‐group (n = 10)	Glargine‐group (n = 10)	*P*‐ value
Number of cats that survived to discharge	8	9	1.000
Time to discharge (h)	172 (91‐221)	140 (51‐173)	**0.033**
Number of cats with spontaneous food intake	9	9	1.000
Time to first spontaneous food intake (h)	54 (6‐132)	26 (12‐54)	0.073
Number of cats with improvement of hyperglycemia (< 1.6% of basal)	10	10	1.000
Time to improvement of hyperglycemia (h)	6 (2‐14)	2 (2‐6)	**0.018**
Number of cats with resolution of hyperglycemia (< 13.9 mmol/L [250 mg/dL])	8	9	1.000
Time to resolution of hyperglycemia (h)	10 (2‐18)	6 (2‐22)	0.266
Decrease of glucose (mmol/L)/h until glucose < 13.9 mmol/L	0.9 (0.4‐4.1)	1.1 (0.6‐3.8)	0.481
Decrease of glucose (mg/dL)/h until glucose < 250 mg/dL	17 (7‐73)	19 (11‐69)	0.481
Number of cats with resolution of acidosis (pH > 7.27)	8	9	1.000
Time to resolution of acidosis (h)	29 (6‐101)	24 (6‐66)	0.255
Number of cats with improvement of ketonemia (< 6.4 % of basal)	10	9	1.000
Time to improvement of ketonemia (h)	12 (6‐36)	12 (6‐36)	0.687
Number of cats with β‐OHB < 2.55 mmol/L	10	9	1.000
Time to β‐OHB < 2.55 mmol/L (h)	42 (18‐102)	30 (12‐42)	0.092
Number of cats with β‐OHB < 2.00 mmol/L	10	9	1.000
Time to β‐OHB < 2.00 mmol/L (h)	42 (18‐108)	30 (12‐42)	0.078
Number of cats with severe or moderate hypothermia (< 36.49°C)*	1	0	1.000
Number of cats with bradycardia (< 120/min)*	0	1	1.000
Lowest glucose (mmol/L)*	10.6 (7.3‐19.9)	10.7 (4.44‐16.9)	0.356
Lowest glucose (mg/dL)*	191 (132‐358)	192 (80‐303)	0.356
Number of cats with hypoglycemia (< 3 mmol/L [< 54 mg/dL])*	0	0	1.000
Highest glucose (mmol/L)*	23.4 (18.4‐31.1)	23.1 (17.5‐33.2)	0.905
Highest glucose (mg/dL)*	421 (332‐561)	416 (315‐598)	0.905
Lowest pH*	7.08 (6.97‐7.31)	7.17 (7.02‐7.33)	0.278
Lowest HCO_3_ (mmol/L)*	9 (5.7‐17.9)	10.6 (7.1‐20.9)	0.447
Lowest potassium (mmol/L)*	2.3 (1.8‐3.2)	2.3 (1.8‐4.4)	1.000
Number of cats with severe hypokalemia (< 3 mmol/L)*	9	8	1.000
Lowest phosphorus (mmol/L)*	0.9 (0.38‐2.41)	0.83 (0.34‐1.9)	0.720
Number of cats with severe hypophosphatemia (< 0.485 mmol/L)*	2	2	1.000
Highest corrected chloride (mmol/L)*	118 (114‐124)	115 (112‐124)	0.356
Number of cats with hyperchloremia (corrected, > 123 mmol/L)*	1	1	1.000
Highest sodium until β‐OHB < 2.55 mmol/L (mmol/L)*	155 (144‐172)	156 (142‐174)	0.720
Number of cats with hypernatremia (> 164 mmol/L)*	1	2	1.000
Δ Weight (kg)	0.1 (‐0.2 to ‐0.3)	0.1 (‐0.1 to ‐0.36)	0.733
Δ HCT (L/L)	‐0.04 (‐0.40 to ‐0.14)	‐0.07 (‐0.15 to ‐0.02)	0.286
Δ Total protein (g/L)	‐7 (‐45 to ‐9)	‐13 (‐33 to ‐6)	0.967
Δ Creatinine (μmol/L)	‐17.7 (‐106.1 to ‐70.7)	‐8.8 (‐44.2 to 17.7)	0.531
Δ Alanine aminotransferase (U/L)	30 (‐483 to ‐758)	87 (‐151 to ‐825)	0.541
Number of IM insulin injections	0	4 (1‐12)	

Δ, difference between baseline and discharge.

Data are presented as number of animals or median (range). *P* < 0.05 is considered significant.

* Untilβ‐OHB < 2.55 mmol/L, basal results not included.

## 4 DISCUSSION

Comparable to the situation in animals, the ideal type of insulin and the optimal route of insulin administration is a matter of debate in human patients with DKA.^18^, ^34^ Except for resource‐poor countries,^17^ currently the most common treatment for human patients is continuous IV infusion of regular insulin because of its rapid onset of action and ease of dose titration.^34^ Nevertheless, labor and cost reductions are considered advantageous in developed countries, especially when patients treated with IV insulin are required to be admitted to an ICU or a specialized diabetes care unit.^35^

The results of our study in cats with naturally occurring DKA corroborate the findings of Marshall et al,^8^ who showed that the basal‐bolus administration of glargine insulin is a safe and effective alternative to the regular insulin CRI protocol currently used by most experts in the field.^3^, ^4^, ^36^, ^37^ Furthermore, as already shown by Gallagher et al,^5^ the application of this simplified basal‐bolus protocol decreased time to improvement of hyperglycemia and to discharge, without affecting survival rate or incidence of adverse events. Although it could be argued that this more rapid decrease in glucose concentrations, likely caused by the higher insulin concentrations achieved within the first hours in the glargine group, would increase the risk of serious complications such as cerebral edema, several studies in dogs and cats suggest that this concern is unwarranted.^36^, ^38^, ^39^ It has been demonstrated in cats with diabetic ketosis that despite significant decreases of blood glucose concentrations > 120% within the first 72 hours of conventional therapy, sodium, the major determinant of serum tonicity, increases and effective osmolality stays relatively constant, minimizing large osmotic shifts.^39^ None of the cats in our study exhibited clinical signs suggestive of hypoglycemia or cerebral edema. Although the final costs of therapy were not calculated, the application of the glargine‐protocol decreased the median time to discharge by almost 1.5 days, which likely translates to substantial cost savings for the owners. In addition, shorter time to first meal and quicker resolution of indicators of DKA could decrease the time in the ICU, further lowering costs to the client. In a large diabetic pet survey including 1192 veterinarians from North America, Europe, and Australia, “costs” were perceived as the second most important motivating factor after “presence of concurrent disease” when owners elected for euthanasia.^40^

DKA treatment is traditionally guided by changes in blood glucose concentrations and blood gas analysis. The results of our study suggest that, as in human patients^41^ and in dogs,^42^ β‐OHB measurements are a useful adjunct for monitoring response to therapy and eliminate the need for frequent blood gas analyses, which are not specific for ketone production, are labor intensive and, therefore, expensive. Additionally, β‐OHB measurements facilitate rapid reevaluation of the diagnosis in cats with low ketone concentrations but ongoing metabolic acidosis. Validated point‐of‐care ketone meters are readily available for cats.^22^, ^43^ ^,£^ The primary endpoint of treatment in our study was the resolution of ketonemia consistent with the definition of DKA, which is a β‐OHB concentration < 2.55 mmol/L. In the absence of significant comorbidities, concentrations below this cut‐off are rarely associated with metabolic acidosis in cats,^22^ and DKA was not diagnosed at values < 3.8 mmol/L in another study.^43^ With the resolution of ketonemia to < 2.55 mmol/L, most of the cats in our study began to eat spontaneously and were transitioned to SC glargine alone. The results of blood gas analysis did not influence insulin dose in any cat.

The median time for resolution of ketonemia in our CRI‐group was 42 hours, compared to 62 and 68 hours in 2 previous studies.^1^, ^5^ A possible explanation is the use of a different protocol for adjustment of insulin and glucose administration. In contrast to the previous studies, insulin infusion was not decreased at glucose concentrations < 16.8 mmol/L (300 mg/dL)^1^ or < 13.9 mmol/L (250 mg/dL),^5^ but insulin administration rate was kept constant until target β‐OHB concentrations were reached. To allow for intensive insulin treatment, 5% glucose solutions were administered when glucose concentrations fell below 10 mmol/L (180 mg/dL). This is in line with the latest treatment guidelines in decompensated diabetic children,^19^ in which a decrease of insulin dose of < 0.05 to 0.1 unit/kg/h is only recommended after DKA has resolved or hypoglycemia is impending, despite the use of 10% or even 12.5% glucose solutions. The early reduction of the CRI‐insulin dose in the cited studies^1^, ^5^ possibly delayed the resolution of ketonemia and could explain why more key indicators were significantly different between the 2 protocols in Gallagher et al's^5^ study.

Despite the fact that initial β‐OHB concentrations of 6.85 mmol/L (4‐8 mmol/L) in our cats are comparable to the concentrations usually seen in human patients with DKA,^44^, ^45^, ^46^ the median (range) times to resolution of ketonemia in the CRI‐ and glargine‐group were 42 (18‐102) and 30 (12‐42) hours, compared to about 11 to 12 hours in people,^46^, ^47^ respectively. Further, a decrease of β‐OHB by more than 1 mmol/L/h, which suggests adequacy of therapy in people,^44^ was not seen in any of our cats irrespective of the protocol used. One explanation is the different etiology of DKA in the 2 species. Whereas type 1 diabetes and insulin omission are the most common causes of DKA in people,^46^ the majority of ketoacidotic cats likely have type 2 diabetes, and many have a concurrent disorder such as acute pancreatitis, hepatic lipidosis, chronic kidney disease, or urinary tract infection.^4^ It is also possible that the higher insulin dose of about 0.1 units/kg/h used in adult human patients is beneficial and accelerates metabolic improvement. Two retrospective studies compared outcome in cats treated with either 0.05 or 0.1 units/kg/h.^4^, ^9^ Whereas the higher insulin dose reduced the odds of poor outcome defined as deaths in the first study,^4^ no differences were found in the second.^9^ Neither of the studies compared time until resolution of ketoacidosis or ketonemia.

A topic of debate in human medicine is whether to routinely start SC administration of a long‐acting basal insulin (eg, glargine insulin) at the onset of DKA management. The rationale is to provide stable background insulin concentration and to avoid reoccurrence of hyperglycemia during the transition time to SC insulin.^18^, ^48^ A common concern raised with SC insulin administration in dehydrated patients is SC insulin accumulation and sudden release after rehydration.^49^ A meta‐analysis of 4 studies in human patients suggests that this concern is likely unwarranted. The addition of SC glargine insulin to standard protocols using IV infusion of regular insulin significantly decreased the time to resolution of DKA, without increasing the risk of adverse events.^50^ Interestingly, Shankar et al^51^ proposed that the positive effects are caused by yet to be described mechanisms, other than just increased total daily insulin dose. Based on evidence in human and feline patients, some veterinary specialists are combining IV regular insulin infusions with twice‐ daily SC glargine insulin injections.^37^ Prospective randomized studies are required to more conclusively estimate the benefit of adding SC insulin glargine to DKA protocols for cats.

Recent research in cats investigated the use of a balanced crystalloid solution in the emergency setting to avoid hypernatremia and excess chloride potentially causing hyperchloremic metabolic acidosis. The use of Normosol‐R in cats with urethral obstruction resulted in more rapid correction of the blood acid‐base disturbance than traditional 0.9% sodium chloride.^52^ Despite the fact that balanced solutions might be advantageous, 0.9% saline was used in the current study as a first‐line treatment because it is still the standard solution in human patients with DKA,^47^ universally available, and inexpensive. Further, like Normosol‐R, which is unavailable in many countries, it is calcium free, allowing the addition of phosphorus‐containing solutions. In contrast to human patients where the incidence of hyperchloremia increases markedly within the first 20 hours of infusion therapy,^53^ hyperchloremia was rarely observed in our study. The most likely explanation is that 85% of our cats were hypochloremic at presentation, whereas this seems to be less common in ketoacidotic human patients.^54^ An earlier study demonstrated that 0.9% saline was effective and safe for volume resuscitation and maintenance fluid therapy in ketoacidotic cats.^1^

To avoid adverse events associated with hypophosphatemia such as hemolytic anemia, IV phosphorus supplementation (potassium‐phosphate) was a fixed part of our treatment protocol. Severe depletion of phosphorus may lead to ATP depletion in erythrocytes, causing failure of actin and myosin fibers in the cell membranes to maintain normal biconcave structure and deformability.^27^ In our protocol, phosphorus was preventatively administered by providing 25% of potassium as potassium‐phosphate. Consequently, hypophosphatemia was observed in only 50% of the cats, compared to 80%,^8^ 67%,^1^ and 65%^4^ in previous studies, in which phosphorus was administered exclusively to hypophosphatemic patients. Consistent with the expected effects of phosphorus depletion on erythrocyte stability, lower phosphorus concentrations were associated with a greater decrease of HCT from baseline to discharge in our study. Noteworthy, of the 4 cats with severe anemia, all had moderate to severe hepatic lipidosis, 3 had documented hypophosphatemia, and 2 had gastrointestinal hemorrhage. It is possible that the “refeeding syndrome,” which has been associated with hepatic lipidosis and characterized by the development of severe hypophosphatemia,^55^ was a causative factor for the development of anemia in some of the cats in the current study.

Several important limitations must be recognized when interpreting the results of our study. The major limitation was that due to stringent criteria for inclusion, the number of cats included was lower than initially planned. A sample size calculation, assuming a power of 80% and a 5% significance level, revealed that a minimum of 37 cats in each treatment group would have been needed to show a 12‐hour difference until resolution of ketonemia. In the future, multisite studies are recommended to provide sufficient power. Other limitations of the study are that the patient population described may not be representative for all cats with DKA, that insulin and infusion requirements and final costs were not compared, that bias may have been introduced by missing data, that it was not possible to definitely diagnose all concurrent disorders, that calcium and magnesium were measured merely in selected cases, that not all cats achieved the measured endpoints, and that it is possible that greater familiarity with the CRI‐protocol, the standard protocol in our clinic before the study, resulted in more proficient implementation than the glargine‐protocol.

In conclusion, although the study was underpowered to detect differences in time until resolution of ketonemia, the results suggest that the basal‐bolus administration of insulin glargine is a useful alternative to the current standard CRI‐protocol for the management of DKA in cats. It is simple and associated with a shorter time to first improvement of hyperglycemia and decreased hospitalization time. Additionally, β‐OHB measurements using hand‐held ketone meters are a useful adjunct for monitoring response to therapy and eliminate the need for frequent blood gas analyses. Further studies are required to evaluate the benefits and disadvantages of IM bolus vs CRI protocols and to compare choices of fluid therapy in management of feline DKA.

## ENDNOTES

a Freestyle Freedome, Abbott Diabetes Care, Alameda, CA.

b Precision Xtra, Abbott Diabetes Care, Alameda, CA.

c Belua, Wellion/MedTrust GmbH, Marz, Austria.

d Malerba E, del Baldo F, Zeppi A, et al. Evaluation of one portable blood glucose meter and one portable glucose‐ketones meter in cats. In: 27th ECVIM‐CA Congress 2017; Abstract, pp. 184.

e Synthesis 25, Instrumentation Laboratories, San Diego, CA.

f Cobas 6000 analyzer series module 501, Roche, Basel, Switzerland.

g Maropitant, Cerenia, Zoetis, Louvain‐la‐Neuve, Belgium.

h Esomeprazole, Esomeprazol Stada 40 mg, Stada, Vienna, Austria.

i Humulin “Lilly” Normal, Eli Lilly, Vienna, Austria.

j Lantus, Sanofi Aventis, Frankfurt, Germany.

k Recovery Liquid, Royal Canin, Vienna, Austria.

l Federico Fracassi, University of Bologna, 2019 (personal communication).

m IBM SPSS Statistics for Windows, version 24.0, IBM, Armonk, NY.
